# Two versus three magnesium screws for osteosynthesis of mandibular condylar head fractures: a finite element analysis

**DOI:** 10.1007/s00784-024-05927-5

**Published:** 2024-09-27

**Authors:** Daphne Schönegg, Günter T. Müller, Michael Blumer, Harald Essig, Maximilian E. H. Wagner

**Affiliations:** 1 Department of Cranio-Maxillofacial Surgery, University Hospital of Bern, Freiburgstrasse 20, Bern, CH-3010 Switzerland; 2 Department of Oral and Maxillofacial Surgery, University Hospital Zurich, Frauenklinikstrasse 24, Zurich, CH-8091 Switzerland

**Keywords:** Finite element analysis, Mandibular condylar head fracture, Osteosynthesis, Von mises stress distribution, Magnesium, Titanium

## Abstract

**Objectives:**

Previous finite element analyses (FEA) have shown promising results for using two titanium screws in treating mandibular condylar head fractures but limited mechanical stability of a two-screw osteosynthesis with magnesium screws. Given the potential benefits of magnesium screws in terms of biocompatibility and resorption, this study aimed to compare two- and three-screw osteosynthesis solutions for a right condylar head fracture (AO CMF type p) with magnesium screws with a FEA.

**Materials and methods:**

A previously validated finite element model simulating a 350 N bite on the contralateral molars was used to analyze von Mises stress within the screws, fragment deformation, and fracture displacement. All screws were modeled with uniform geometric specifications mirroring the design of Medartis MODUS^®^ Mandible Hexadrive cortical screws.

**Results:**

The three-screw configuration demonstrated lower values for all three parameters compared to the two-screw scenario. There was a 30% reduction in maximum von Mises stress for the top screw and a 46% reduction for the bottom screw.

**Conclusions:**

Fracture treatment with three magnesium screws could be a valuable and sufficiently stable alternative to the established treatment with titanium screws. Further studies on screw geometry could help improve material stability under mechanical loading, enhancing the performance of magnesium screws in clinical applications.

**Clinical relevance:**

The use of magnesium screws for osteosynthesis of mandibular condylar head fractures offers the benefit of reducing the need for second surgery for hardware removal. Clinical data is needed to determine whether the advantages of resorbable screw materials outweigh potential drawbacks in condylar head fracture treatment.

## Introduction

Open reduction and internal fixation (ORIF) of mandibular condylar head fractures might improve functional outcomes compared to closed treatment and is therefore suggested for selected cases [1, 2]. It typically involves the insertion of titanium screws through an extraoral approach [3, 4]. Resorbable magnesium screws have emerged as a promising alternative due to their favorable biomechanical behavior and their potential to avoid the need for a second surgery for screw removal as they gradually resorb over time [5, 6]. This might be particularly advantageous in patients where long-term retention of metallic implants or hardware removal are undesirable.

A previous finite element analysis (FEA) slightly favored two-screw osteosynthesis with titanium screws over magnesium screws [7] for osteosynthesis of an AO CMF type p condylar head fracture. This study aimed to assess whether osteosynthesis of a mandibular head fracture with three magnesium screws shows more desirable results regarding fragment deformation, fracture displacement, and tensile stress.

## Materials and methods

For this study, a previously validated FEA model of a mandible with a right-sided AO CMF type p condylar head fracture was used, as detailed in Schönegg et al. [7, 8]. Load boundaries based on the validated model by de Zee et al. [9] were applied.

All screws were modeled to be of a magnesium alloy with an elastic modulus of 44,100 MPa and a Poisson’s ratio of 0.27. Uniform geometric specifications were applied, mirroring the design of Medartis MODUS^®^ Mandible Hexadrive 1.8 mm cortical screws measuring 13 mm in length (Medartis AG, Basel, Switzerland).

The same loading scenario simulating biting on the contralateral (left) molars with 350 N was calculated for osteosynthesis with two versus three magnesium screws. A qualitative analysis of differences in von Mises stress distribution within each screw, fragment deformation, and fracture displacement was performed.

## Results

### Fragment deformation and fracture displacement

**Fig. 1:**
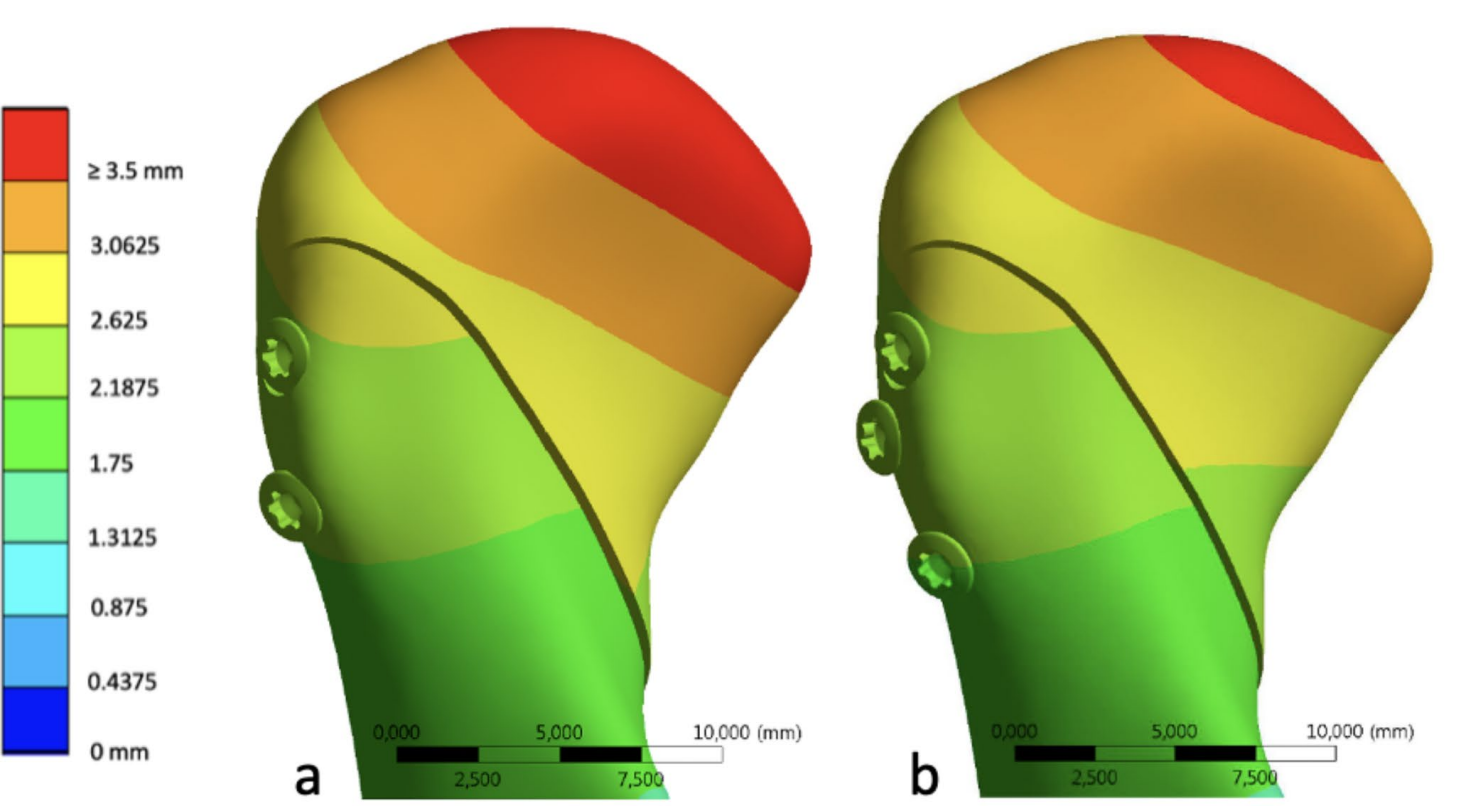
Comparison of fragment deformation for two (**a**) versus three (**b**) screws

Fracture displacement was found to be similar in both osteosynthesis scenarios, albeit with a slightly smaller opening of the fracture gap towards the anterior in the three-screw configuration. Furthermore, maximum fragment deformation showed a decrease with three screws, measuring 3.6 mm as opposed to 3.9 mm at the tip of the condylar head (Fig. 1).

### Von mises stress distribution

**Fig. 2:**
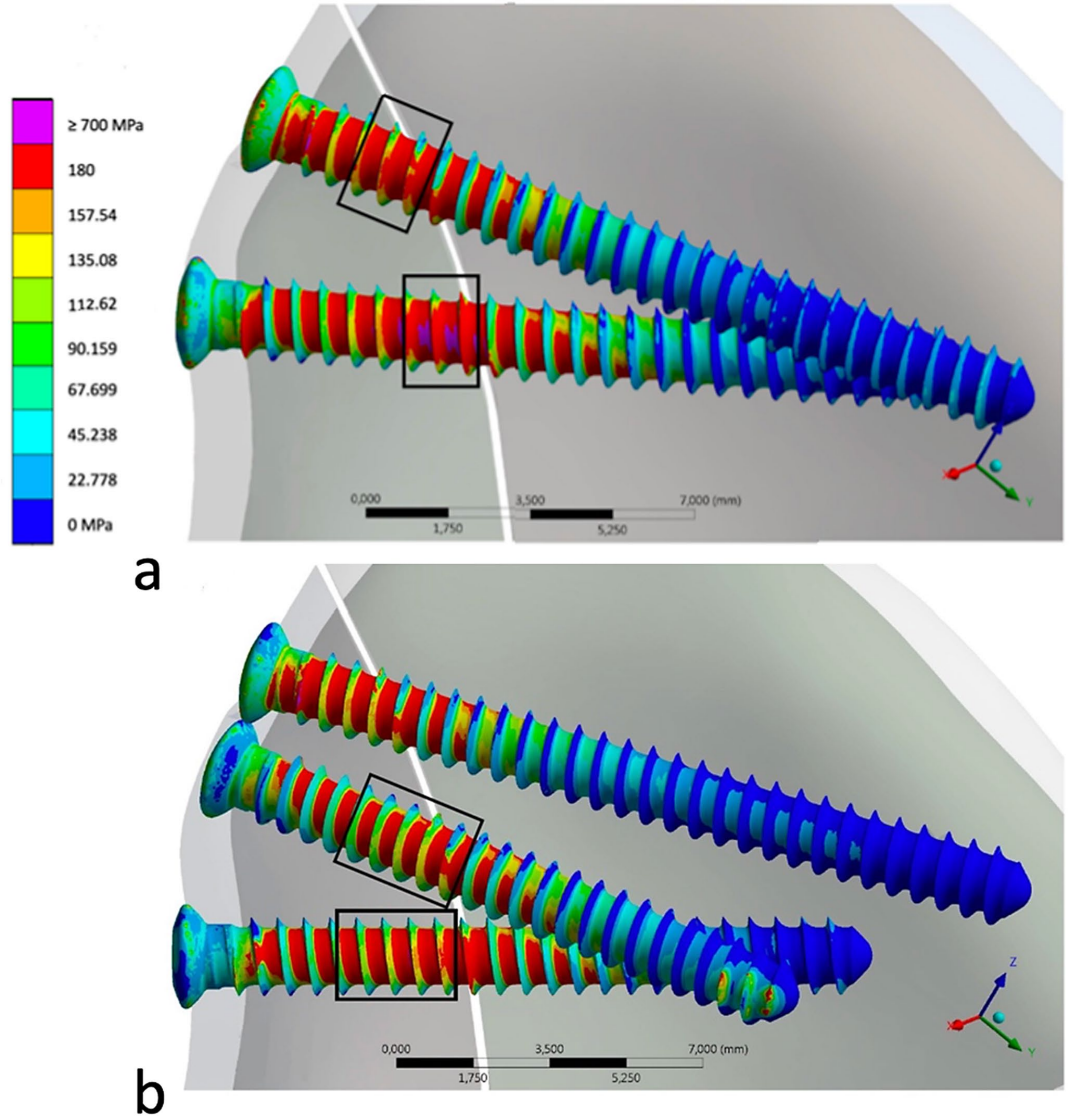
Comparative analysis of the von Mises stress distribution in the two-screw (**a**) and three-screw (**b**) scenarios. Areas where tensile strength is exceeded are shown in pink

As illustrated in Fig. 2, the highest von Mises stress was localized within the fracture gap region, specifically concentrated on the bottom screw within the lateral fracture fragment in both the two- and three-screw scenarios. In the latter, a secondary area of heightened von Mises stress was identified at the tip of the middle screw in the medial fragment.

**Fig. 3:**
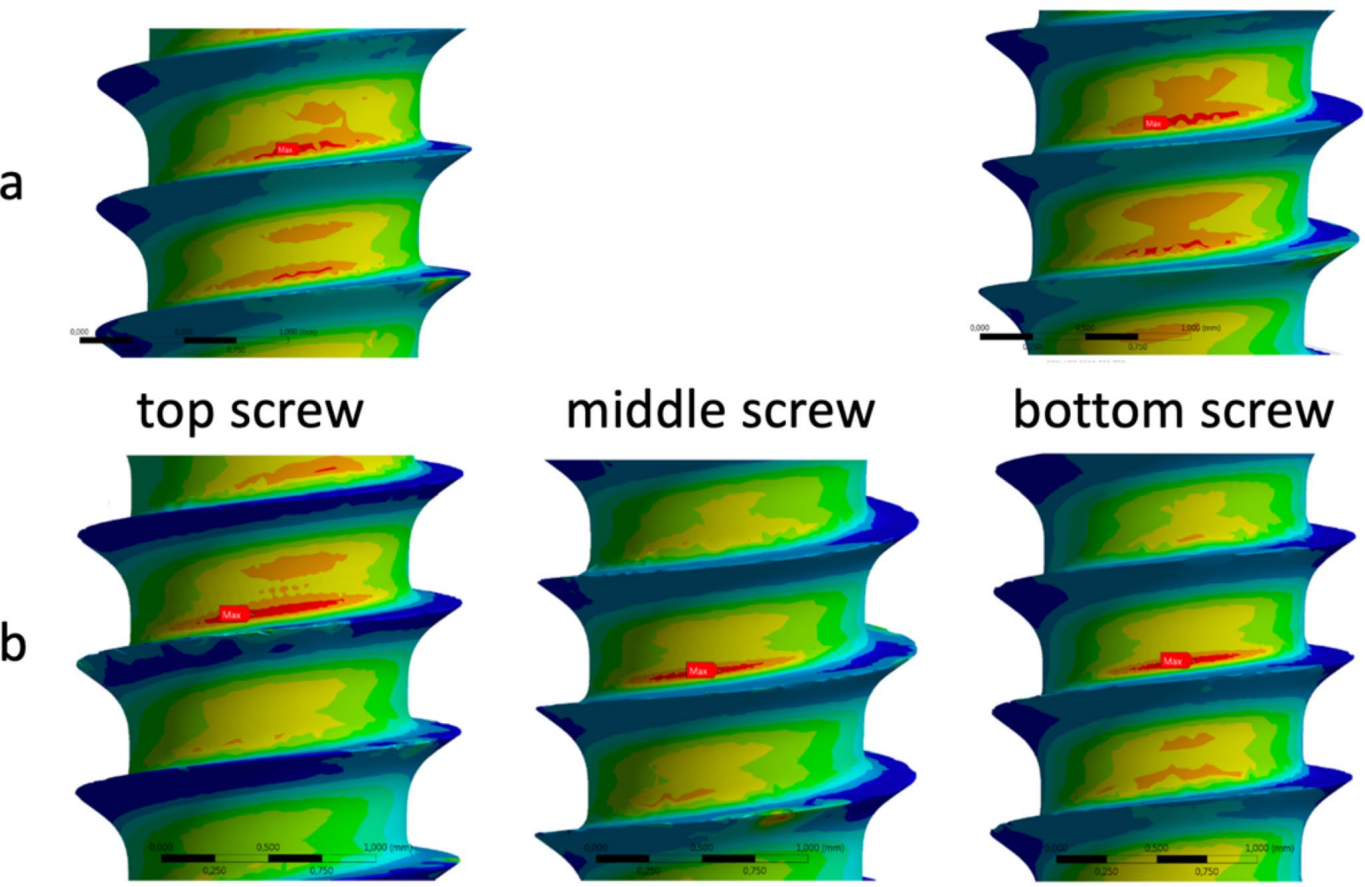
Maximum von Mises stress is concentrated at the root of the screws in the region of the fracture gap in the two-screw (**a**) and three-screw (**b**) scenarios

Detailed analysis of the von Mises stress within the screws in the region of the fracture gap showed a consistent concentration at the root of the screw in all screws (Fig. 3).

The maximum von Mises stress exceeded the material’s tensile strength in the bottom screw of the two-screw configuration with a peak value of 1373 MPa. In the three-screw scenario, in contrast, the maximum stress values did not surpass 700 MPa.

In the three-screw scenario, the maximum von Mises stress experienced a reduction of 30% for the top screw and 46% for the bottom screw in comparison to the two-screw scenario. Despite still surpassing the material’s tensile strength by 333%, this represents a substantial improvement when contrasted with the 479% exceedance observed in the two-screw configuration.

## Discussion

When planning open reduction and internal fixation (ORIF) of a mandibular condylar head fracture, maxillofacial surgeons are tasked not only with the imperative of achieving biomechanical stability but also with the intricacies of managing biological tissues and subsequent postoperative processes. Factors impacting both aspects include the choice of screw material – whether titanium or a resorbable alternative – and the quantity of screws employed. Factors to consider when choosing the osteosynthesis material include the risk of increased osseous remodeling after multiple drilling and exerting forces through screw heads, the complications associated with the surgical approach, and the necessity for repeat procedures for hardware removal [10]. Additionally, consideration must be given to the resorption chemistry of bioresorbable materials [11, 12].

The advantages and drawbacks of the finite element model employed in this study have been discussed in prior works [7, 8]. A load of 350 N on the contralateral molars was selected, which approaches the upper limit of physiological bite forces. While this choice facilitates the visualization of forces, stress, and fragment displacement, it is essential to recognize that these forces correspond to an extreme scenario where osteosynthesis failure is likely. Considering that patients typically adhere to a soft diet postoperatively, this situation may not fully reflect the usual clinical conditions. Nevertheless, a qualitative analysis of the outcomes derived from this model is justified and can contribute to a better understanding of the biomechanical behavior of a magnesium alloy screw before applying it clinically.

Differences in fracture displacement and fragment deformation between the two- and three-screw scenarios were found to be small, but noticeable in this analysis, suggesting that while osteosynthesis with two magnesium screws might provide sufficient stability in a clinical scenario, the three-screw solution still offers a more stable option. It is noteworthy that the material’s tensile strength was exceeded by 479% in the two-screw scenario, a significantly higher margin compared to the application of three screws. Clinically, this could potentially result in osteosynthesis failure due to either screw loosening or fracture. Furthermore, the von Mises stress on the screws in the region of the fracture gap experienced a notable reduction when the forces were distributed among three screws. Improvement of biomechanical stability by increased numbers of magnesium screws has also been demonstrated by other authors [13]. Interestingly, a second von Mises stress maximum was observed at the tip of the middle screw, possibly indicative of the complex three-dimensional force pattern effective in the condylar head. This is in accordance with a study examining the influence of screw placement geometry on resulting von Mises stress distribution and fracture dislocation [14]. All screws were modeled to mirror the design of Medartis MODUS^®^ titanium screws in this FEA study, as this is the screw type most used for ORIF of mandibular condylar head fractures in our university hospital’s maxillofacial surgery department. Adaptation in screw geometry could also positively affect the biomechanical stability of screws fabricated from a magnesium alloy and will have to be examined in a further study.

The findings of this finite element analysis suggest the use of three magnesium screws to enhance biomechanical stability in the osteosynthesis of mandibular condylar head fractures. Further studies, preferably prospective clinical trials, are needed to clarify whether the benefits derived from the heightened biomechanical stability achieved with three magnesium screws outweigh the drawbacks associated with increased drilling and foreign material. Based on clinical experience in the authors’ department, the length of surgery, overall cost, surgeon satisfaction with fragment repositioning and functional outcomes are similar when using two or three titanium screws. Ultimately, the decision on the number and type of osteosynthesis screws should be approached on a patient-specific basis. Case-by-case evaluation, considering factors such as the fracture pattern, patient characteristics, surgeon expertise, and the materials available at a given facility will enable the proper assessment of whether the above-mentioned advantages translate into significant clinical benefits for each unique case.

## Conclusion

Resorbable magnesium screws offer a useful addition to the osteosynthesis repertoire for mandibular condylar head fractures. The findings of this FEA indicate that the insertion of three magnesium screws is advisable for attaining biomechanically stable osteosynthesis. Further studies focusing on the biological effects of two- versus three-screw osteosynthesis will help elucidate the clinical implications of different ORIF techniques. Adaptation of the screw geometry and optimization of the screw placement geometry could also open up further possibilities for mandibular condylar head fracture treatment.

## Data Availability

No datasets were generated or analysed during the current study.
